# Construction of geriatric hypoalbuminemia predicting model for hypoalbuminemia patients with and without pneumonia and explainability analysis

**DOI:** 10.3389/fmed.2024.1518222

**Published:** 2024-12-31

**Authors:** Ziqi Liu, Qi Kang, Zhilong Mi, Yuan Yuan, Tiantian Sang, Binghui Guo, Zhiming Zheng, Ziqiao Yin, Wei Tian

**Affiliations:** ^1^School of Mathematical Sciences, Beihang University, Beijing, China; ^2^Key Laboratory of Mathematics, Informatics and Behavioral Semantics and State Key Laboratory of Software Development Environment, Beihang University, Beijing, China; ^3^Department of Geriatrics, Beijing Jishuitan Hospital, Capital Medical University, Beijing, China; ^4^Institute of Artificial Intelligence, Beijing Advanced Innovation Center for Future Blockchain and Privacy Computing, Beihang University, Beijing, China; ^5^Zhongguancun Laboratory, Beijing, China; ^6^Hangzhou Internation Innovation Institute of Beihang University, Hangzhou, China

**Keywords:** geriatric, pneumonia, hypoalbuminemia, statistical analysis, machine learning prediction, gradual fusion prediction

## Abstract

**Background and objectives:**

Pneumonia portrays a critical health concern in geriatrics. Geriatric pneumonia can lead to changes on other complications, in which hypoalbuminemia is a common complication. However, few studies have looked at the impact of pneumonia on the course of hypoalbuminemia and predicting. This study aims to predicting hypoalbuminemia in geriatric pneumonia and non-pneumonia patients and exploring the clinical difference between the two groups.

**Materials and methods:**

This retrospective study enrolled 42 pneumonia patients group and 76 non-pneumonia patients group. The indicators difference of different groups were analyzed, then a mutual information-grey relational coefficient gradual fusion model was constructed to predict hypoalbuminemia in the future by the indicators of vital signs, N-Terminal Pro-Brain Natriuretic Peptide, blood routine examination and urine routine examination at admission. Through the sensitivity analysis of model, we analysed the important of four examines in patients with and without pneumonia.

**Results:**

The predicted accuracy of our gradual fusion model was 0.954, which improve the prediction accuracy by nearly 17.6% compared with the classical machine learning method. The AUC of gradual fusion model was 0.96 and 0.9 in hypoalbuminemia patients with and without pneumonia. The sensitivity analysis of gradual fusion model showed blood routine examine was most important to predict hypoalbuminemia in patients with pneumonia, while urine routine examine was most important to predict hypoalbuminemia in non-pneumonia patients.

**Conclusion:**

The changes in the blood of patients with hypoalbuminemia combined with pneumonia were more significant than that of patients with hypoalbuminemia alone, which was characterized by abnormal excretion due to low protein. We suggested doctors should pay more attention to blood routine results when preventing hypoalbuminemia in patients with pneumonia and pay more attention to urine routine examine results when preventing hypoalbuminemia in patients without pneumonia.

## 1 Introduction

Hypoalbuminemia is a common complication of many clinical malignant diseases, such as pneumonia, cardiovascular diseases, diabetes, sepsis, etc., especially in the geriatric population (1–4). Clinically, the index of serum protein is used to measure hypoalbuminemia. Generally speaking, the serum egg value of healthy adults is 35-50 g/L, and hypoalbuminemia is diagnosed when the serum protein value is less than 30 g/L. (5) Studies have shown that for every 10 g/L decrease in serum protein concentration, the mortality rate of patients will increase by 137%, the length of stay in intensive care unit will increase by 28%, and the length of stay will increase by 71% (6). In addition, the plasma albumin level of the geriatric is low due to physiological reasons of age, and the plasma albumin can regulate inflammatory response and play a certain anti-inflammatory role. Therefore, the hypoalbuminemia of the geriatric with acute and chronic infectious diseases will aggravate the severity of the original infectious diseases of the geriatric, affect the prognosis of the disease, and increase the risk of death (7, 8).

Due to the influence of the COVID-19 epidemic in recent years, some scholars have studied the relationship between COVID-19 and hypoalbuminemia, and studies have shown that many COVID-19 patients have hypoalbuminemia, and hypoalbuminemia will aggravate the reaction related to COVID-19 and lead to death (9–12). Now, Many variants of the COVID-19 and even different types of pneumonia have emerged, some study have shown that hypoalbuminemia was a risk factor of pneumonia and the mortality rate is high (13, 14). Other researches on hypoalbuminemia focuses on the analysis of influencing factors of hypoalbuminemia and the prediction of hypoalbuminemia. As for the analysis of influencing factors of hypoalbuminemia, most study evaluated the risk factors associated with hypoalbuminemia by using a typical regression model of multi-factor logistic regression (15–26). There are few studies on the prediction of hypoalbuminemia, most of which use the lasso regression to screen risk factors, and then build a nomal model to predict hypoalbuminemia (27–34). There are some studies using machine learning and data fusion to predict hypoalbuminemia, but the results have not been very good (35, 36).

We believe that the reason for above phenomenon is that current studies have not paid attention to the intrinsic differences between hypoalbuminemia patients with pneumonia and those without pneumonia. So we present a study which aimed at constructing an effective hypoalbuminemia predictive model, further analyzing the difference between hypoalbuminemia patients with and without pneumonia from the aspect of examination upon admission to hospital.

## 2 Materials and methods

### 2.1 Study population

A total of 118 patients hospitalized in Beijing Jishuitan Hospital from January 2021 to January 2022 were included in this study, in which 21 pneumonia with hypoalbuminemia patients (PHPs), 15 non-pneumonia with hypoalbuminemia patients (NPHPs), 21 pneumonia with non-hypoalbuminemia patients (PNHPs), and 61 neither pneumonia or hypoalbuminemia patients (NPNHPs). All cases of hypoalbuminemia occurred within 14 days after hospital admission. Patients with cancer, immune dysfunction, acquired immunodeficiency diseases, hematological disorders, long-term use of immunosuppressants or glucocorticoids, or acute inflammation other than pneumonia were excluded from the study.

In the 118 patients, 55 were males and 63 were females, with an average age of 78.37 ± 8.57 years. The vital signs (VS), N-Terminal Pro-Brain Natriuretic Peptide (NT-proBNP), blood routine examination (BRE) and urine routine examination (URE) of these patients were complete at admission, the VS included respiratory rate (RR) and systolic blood pressure (SBP), the NT-proBNP test included NT-proBNP value. BRE include platelet count (PLT), red blood cell count (RBC), white blood cell count (WBC), red blood cell distribution width-CV (RDW-CV), red blood cell distribution width-SD (RDW-SD), mean red blood cell hemoglobin concentration (MCHC), mean corpsular hemoglobin (MCH), mean corpuscular volume (MCV), hematocrit (HCT), hemoglobin (HB), URE include urine specific gravity (USG), urine Ph (UPh), urine white blood cells (UWBC), urine red blood cells (URBC), equivalent to red blood cells (EQRWC), equivalent to white blood cells (EQWBC). All examination results were collected simultaneously during the early days of the patients’ hospital admission.

Due to the small sample size and the imbalance between the pneumonia patients group and the non-pneumonia patients group with hypoalbuminemia, we performed a simple data enhancement. We standardized the current 118 samples and copied them twice, added the Gaussian noise to the copied samples, and sampled according to the label ratio of pneumonia to hypoalbuminemia. Finally, 108 hypoalbuminemia patients (HPs) and 108 non-hypoalbuminemia patients (NHPs) were obtained, including 63 PHPs, 45 NPHPs, 55 PNHPs, and 53 NPNHPs.

This study was reviewed and approved by the Ethics Review Committee of Beijing Jishuitan Hospital affiliated to Capital Medical University (Ethics approval number: 202106-43-BI 01).

### 2.2 Statistical method

We used the student *T*-test to assess the mean differences in characteristics between the hypoalbuminemia patients and non-hypoalbuminemia patients to understand the differences between pneumonia patients groups and non-pneumonia patients groups. Then, we used logistic regression to establish a regression model for the hypoalbuminemia label of the four test features, and obtained the importance of each test for hypoalbuminemia by observing the weight of the features (37). Pearson correlation coefficient was used to measure the correlation between features, and mantel test was used to analyze the correlation between features and hypoalbuminemia labels (38, 39).

### 2.3 Machine learning method

Since most of the existing studies on hypoalbuminemia are based on regression models, and there are few studies on the prediction task using machine learning models, we use the relatively typical support vector machine and decision tree models in machine learning to complete the prediction task of hypoalbuminemia. Support vector machine is used with linear kernel and radial basis function kernel. General linear support vector machine is mostly used when the feature is linearly separable. After adding radial basis function kernel, its nonlinear mapping ability can map nonlinear problems in low-dimensional space to high-dimensional space, enhancing the divisibility of identified objects (40). For decision tree algorithm, we use a decision tree based on Gini value (41). To prevent overfitting of model training, we set the depth of the tree to a maximum of 4.

### 2.4 Mutual information and grey relational coefficient gradual fusion model

**Figure 1:**
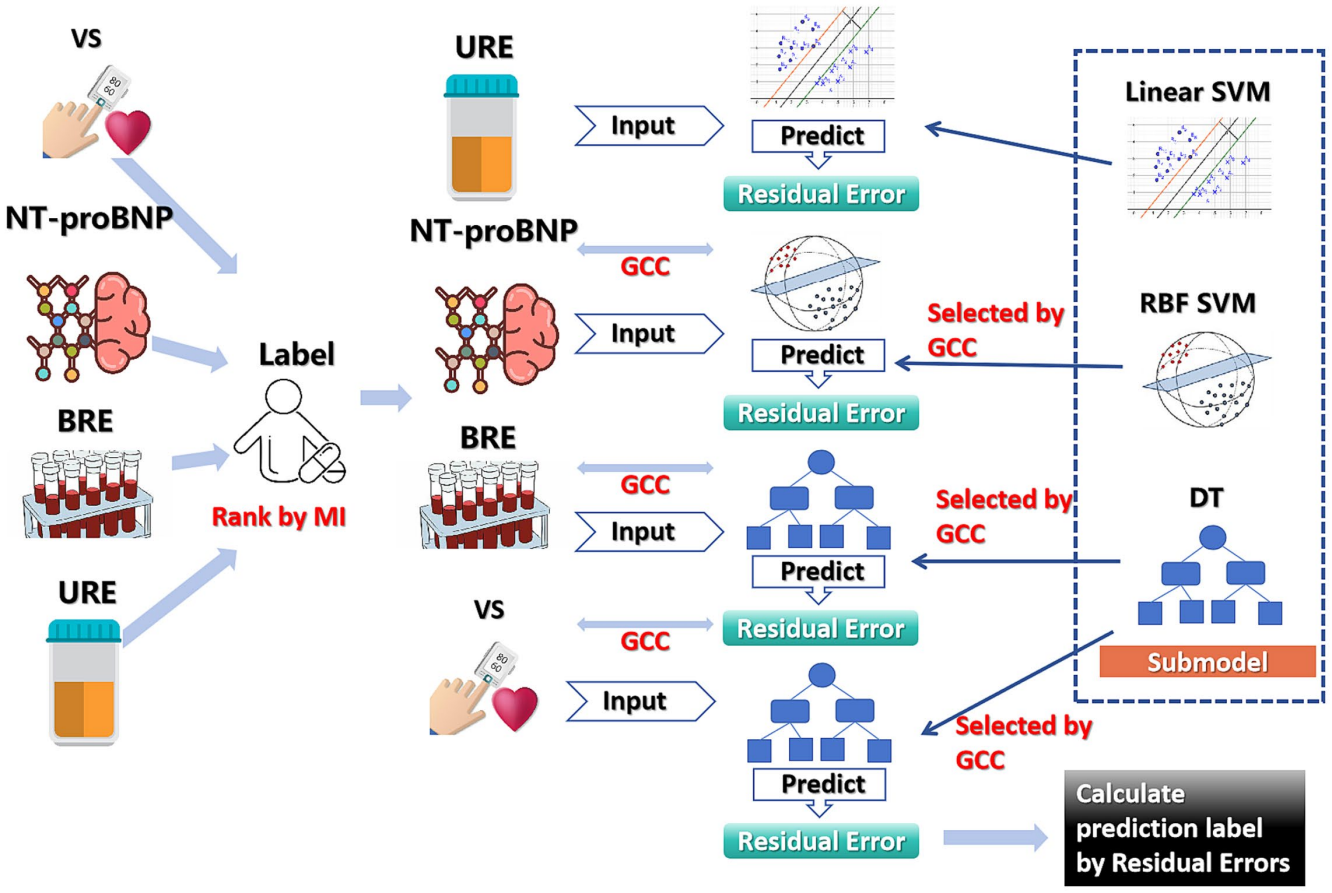
MGGF construction diagram.

In order to explore whether the internal sequential correlation between the test results of patients can improve the prediction accuracy of hypoalbuminemia, we proposed a mutual information and grey relational coefficient gradual fusion (MGGF) model, which uses different examine data step by step according to the average mutual information size between the features contained in the data and the labels. The submodel of each step is determined by the residual predicted in the previous step and the average grey correlation coefficient of the currently used data features. See Supplementary material for detailed construction steps of the model. The model construction flow chart is shown in Figure 1.

## 3 Results

### 3.1 Comparison indicator differences between pneumonia with hypoalbuminemia patients and non-pneumonia with hypoalbuminemia patients

**Figure 2:**
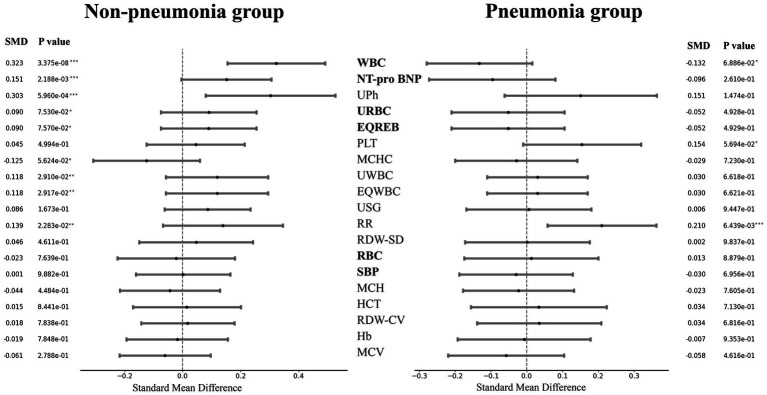
Forest plot of indicators standard mean difference in pneumonia group and non-pneumonia group. The ‘*’ in the *p* value represented significance, ‘***’ represented particularly significant, ‘**’ represented significant and ‘*’ represented a little significant. The bold indicator name indicated that the SMDs of the two groups are different symbols.

Considering that pneumonia would have a certain impact on the onset and even the post-onset course of hypoalbuminemia, we divided patients into pneumonia patients and non-pneumonia patients according to case information. First, we used student T-test to test the difference in characteristics of patients with hypoalbuminemia and non-hypoalbuminemia under the two conditions, and the results are shown in Tables A1, A2. Meanwhile, we present a forest plot of the standardized mean differences (SMD) of indicators between the pneumonia group and the non-pneumonia group, saw Figure 2. The SMD and corresponding *p* values for the pneumonia and non-pneumonia groups are displayed on the right and left sides, respectively. The indicators are arranged from top to bottom based on the magnitude of the SMD difference between the two groups. The results showed that the different indicators of patients with and without hypoalbuminemia presented different results in the premise of with pneumonia and without pneumonia.

**Figure 3:**
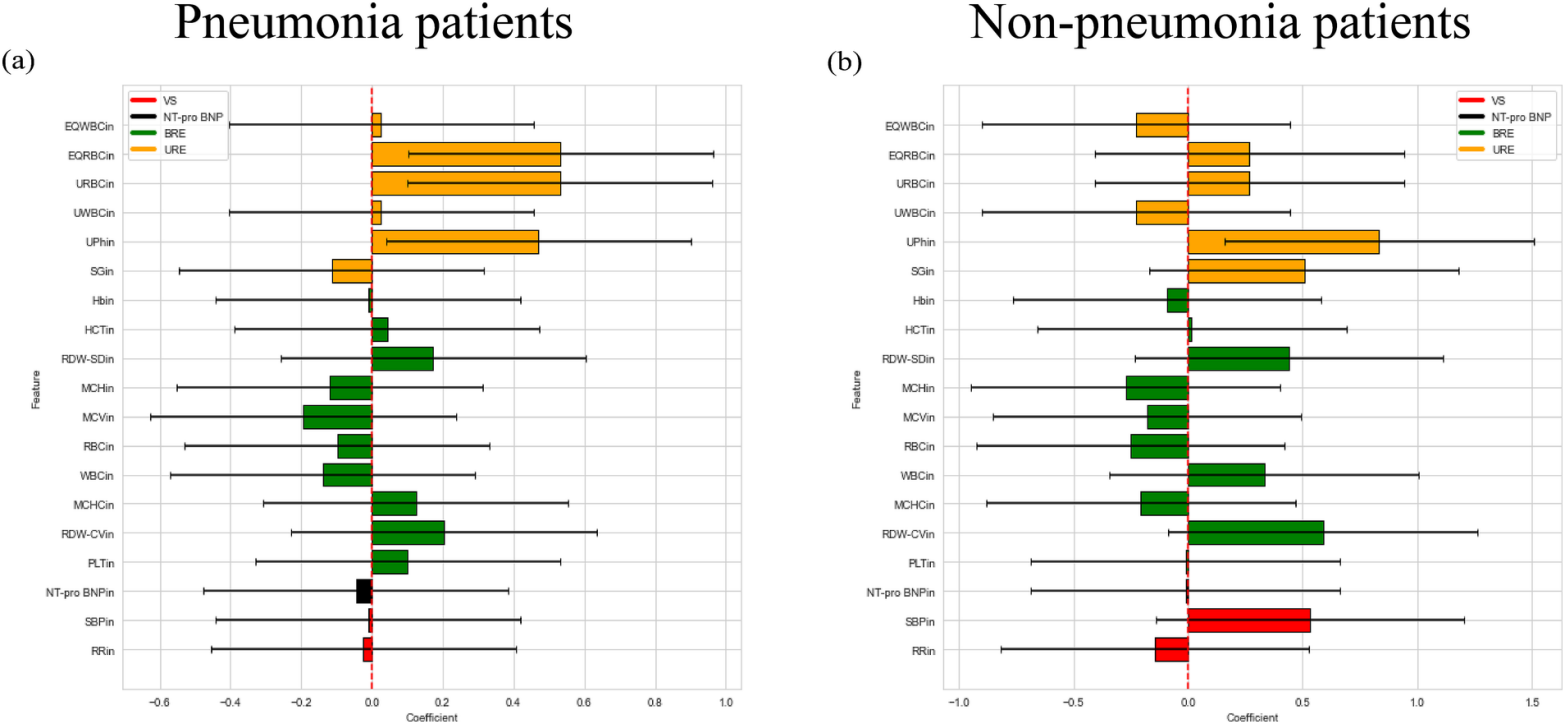
Logistic regression weight plot of patients with and without pneumonia. **(a)** Logistic regression weight plot of pneumonia patients. **(b)** Logistic regression weight plot of non-pneumonia patients.

Then, we used logistic regression to construct regression model of the hypoalbuminemia label with patient indicators in both pneumonia and non-pneumonia conditions, and the results were shown in Figure 3. The average weight of the four examines for the pneumonia patients was 0.3385, 0.011, 0.2386, 0.388, for the non-pneumonia patients was 0.0175, 0.046, 0.121, 0.283. It can be found that the average weight of urine routine examine in patients with and without pneumonia is the highest, the blood routine examine ranked 3th and 2th, respectively.

We also followed the same steps to analyze the difference in indicators and the results of regression analysis when patients did not distinguish whether there was pneumonia or not. See Supplementary material for detailed results.

### 3.2 Results of predicting hypoalbuminemia with pneumonia patients and non-pneumonia patients

**Figure 4:**
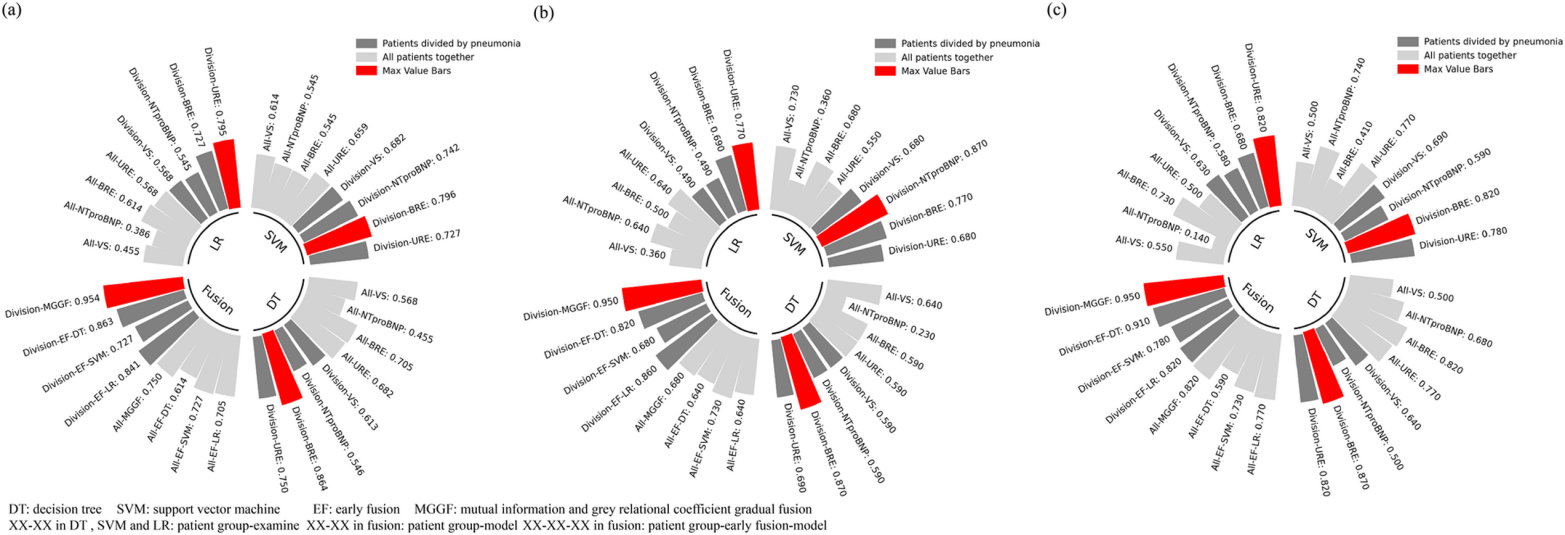
Hypoalbuminemia prediction results. The MGGF model performed best in three evaluation indexes and the prediction effect of models has been significantly improved after patients were divided. **(a)** Accuracy of all prediction models for different patients. **(b)** Recall of all prediction models for different patients. **(c)** Specificity of all prediction models for different patients.

**Figure 5:**
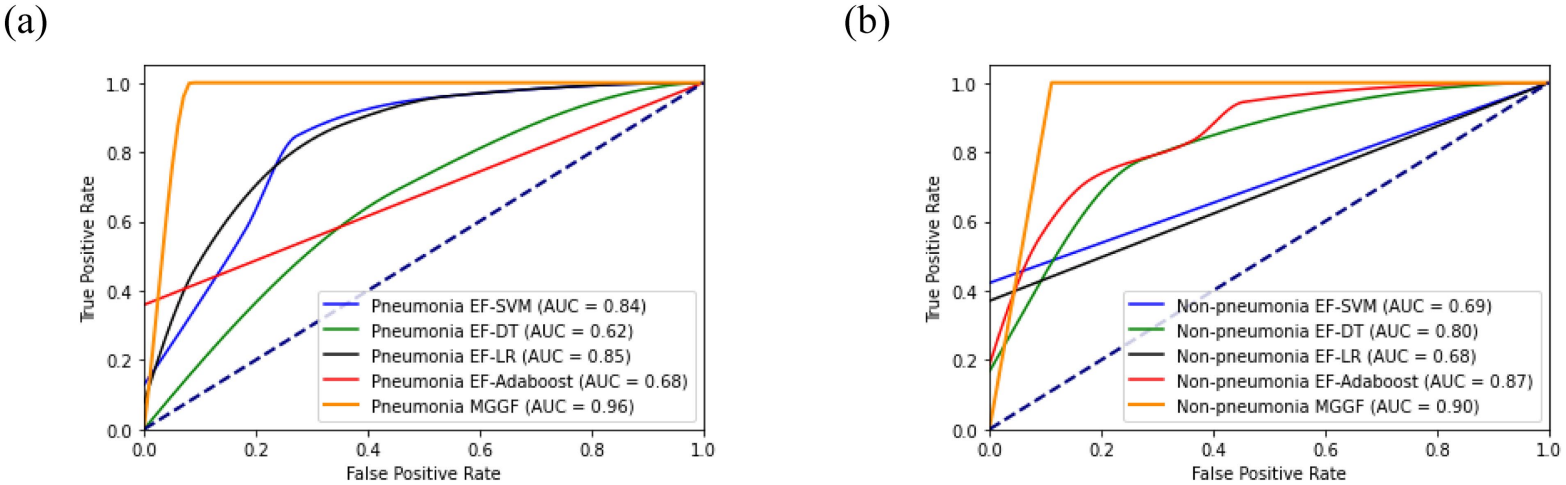
The ROC curve of four EF models and MGGF models in two categories patients. The AUC of MGGF were better than early fusion models in two cases. **(a)** The ROC curve of models in pneumonia patiens. **(b)** The ROC curve of models in non-pneumonia patiens.

We divided patient into pneumonia group and non-pneumonia group. All models was trained on the two groups and then calculated the accuracy, recall and specificity overall. We first trained the support vector machine and decision tree according to the training set and the test set 4:1, respectively. At the same time, experiments on all patients was conducted as a contrast. We further compared the results of early fusion and gradual fusion between all patients and the two groups. The results of mutual information and gray association coefficients in building MGGF were all shown in Supplementary material. All prediction accuracy were shown in Figure 4A, all prediction recall were shown in Figure 4B and all prediction specificity were shown in Figure 4C. We could see that the MGGF model performed best in three evaluation indexes, even the accuracy of MGGF based on two categories patients achieved 0.954. What’s more, the prediction effect of the model has been significantly improved after patients were divided, which may because the interference of redundant information on the model was reduced after the differentiation of patients, making the model prediction more accurate. In order to study the effect of the model in two categories patients, we further compared the ROC curves of the early fusion models and the MGGF model, seen Figure 5. The AUC of MGGF achieved 0.9 in both cases, which were better than early fusion models. It further demonstrated the validity of MGGF model.

**Figure 6:**
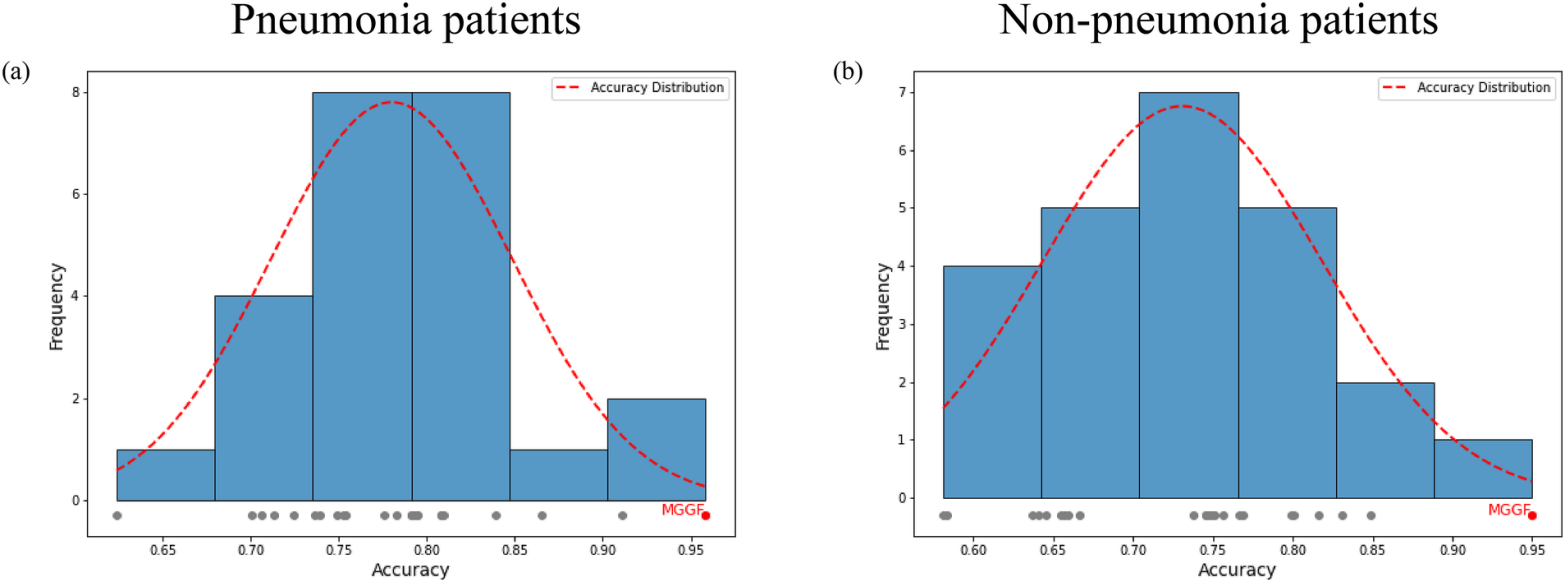
The prediction accuracy of gradual fusion with all input data orders for patients with and without pneumonia. The gray points represented the accuracy of different input data order and the red point represented the highest one. The red curve is the accuracy frequency distribution curve for each interval. The MGGF model was the best in all input data orders. **(a)** The prediction accuracy of gradual fusion with all input data orders for pneumonia patients. **(b)** The prediction accuracy of gradual fusion with all input data orders for non-pneumonia patients.

We also compared the gradual fusion prediction results of all 24 input orders of the four test data, as shown in Figure 6. The red points represents the highest result in all results. The results show that the MGGF is still the best in both cases. The gradual fusion prediction results of all 24 input orders in all patients saw Supplementary material.

### 3.3 The importance of four examines to predicting hypoalbuminemia

We estimate the importance of different examines by performing sensitivity analysis on the gradual fusion model, and then analyze the necessity of performing four tests at the time of admission. We eliminated the four tests in turn, and only used the remaining three tests for gradual fusion. The predicted results are shown in Table 1. We used the accuracy reduction to evaluate the importance of each test under different circumstances. According to Table 1, the prediction accuracy obtained by removing the data of any test was reduced to varying degrees except when clinical features were removed to predict the absence of pneumonia combined with hypoalbuminemia, indicating that these four tests have complementary effects in predicting hypoalbuminemia. In addition, in the case of pneumonia, the removal accuracy of blood routine examine decreased the most. In the absence of pneumonia, the removal accuracy of urine routine decreased the most.

**Table 1:** The prediction results of removing a test.

	VS		NT-proBNP		BRE		URE	
	PWH	NPWH	PWH	NPWH	PWH	NPWH	PWH	NPWH
ACC	0.791	0.95	0.917	0.85	0.75	0.75	0.875	0.7
Recall	0.909	0.91	0.909	0.818	0.727	0.818	0.909	0.909
Specificity	0.923	1	0.923	0.889	0.769	0.667	0.846	0.444
ACC^−^	0.167	-^*^	0.041	0.1	0.208	0.2	0.083	0.25

* The accuracy achieved highest before the examine was used when predicting NPWH.

## 4 Discussion

Patients with hypoalbuminemia have a low concentration of albumin in the blood, and albumin is a substance that enhances immunity and resistance, so pneumonia patients with hypoalbuminemia will affect the cure of pneumonia, and even increase the risk of death (42). If the future hypoalbuminemia can be predicted at an early stage, the exacerbation of the pneumonia will be avoided and the mortality will be reduced. At present, there were some studies on the prediction of hypoalbuminemia, For instance, Wang et al. (33) compared the effect of logistic regression, random forest, support vector machine, extreme gradient enhancement algorithm (XGB) on predicting hypoalbuminemia using demographic characteristics, vital signs, laboratory examination. In the results of their study, support vector machine was 81.13% best in accuracy and logistic regression was 0.8077 best in AUC. In addition Li et al. (35) combined image data and clinical data, and used scores and clinical features for prediction through early fusion, the AUC of his model was 0.8704. However, when many geriatric diseases are combined, they affect each other’s course. The current prediction models of early hypoalbuminemia did not take into account the effects between the diseases, but treat them in a uniform way. We believe that this is one reason why the current prediction of hypoalbuminemia is not good. So that in our study, patients with hypoalbuminemia were divided into pneumonia group and non-pneumonia group.

First, we conducted a difference analysis on the examine indicators of pneumonia patients with and without hypoalbuminemia, and non-pneumonia patients with and without hypoalbuminemia respectively, so as to evaluate the difference between the two groups of people in what aspects of the body. The results showed that the difference indicators between the two groups of people were different, the difference indicators mainly concentrated in blood routine examine in pneumonia patients, this view is similar to the conclusion obtained in Liu’s study (43). But difference indicators mainly concentrated in urine routine examine in non-pneumonia patients, Serebruany’s study has suggested that hypoalbuminemia may be a risk factor in the development of renal failure which was a support to our results (44). Our results indicated that pneumonia maybe affect the pathogenesis of patients with hypoalbuminemia. Then, we built a regression model of the examine indicators on hypoalbuminemia label by using the logistic regression model, obtained the weight of the examine indicators on hypoalbuminemia, and then calculated the average weight of the four examines on hypoalbuminemia. The results showed that the highest weight was urine routine examine in both pneumonia patients and non-pneumonia patients, the blood routine examine was 3th and 2th, respectively. These further demonstrated that the blood routine examine and urine routine examine were two examines with a certain degree of significance.

In order to further improve the accuracy of prediction, we introduce two typical machine learning, SVM and DT models machine learning algorithm, to predict on the basis of traditional statistical methods. Considering the results of difference analysis, we predicted hypoalbuminemia in two groups, respectively. Most traditional machine learning algorithms are based on the assumption that features are independent of each other. However when diagnosing complex diseases, patients’ various examine indicators are not independent of each other, just as doctors consider the results of different examines synthetically when diagnosing a disease, and then giving the finally conclusion (45, 46). Moreover, different examinations for a single patient can be viewed as analyzing a complex system from various perspectives. The effects of different diseases may cause abnormalities in specific tissues or organs, which, in turn, can lead to secondary abnormalities in other tissues and organs. As a result, examinations may exhibit distinct internal sequential correlations within different disease groups. Statistical analysis in our study further demonstrated that pneumonia influences changes in certain indicators associated with hypoalbuminemia, thereby impacting their predictive effectiveness for hypoalbuminemia. While traditional machine learning approaches treat all indicators as equally significant, our model introduces a progressive fusion approach. By leveraging mutual information entropy, we quantify the predictive value of indicators for hypoalbuminemia under the influence of pneumonia. This enables a stepwise prediction of hypoalbuminemia risk using these indicators. Additionally, we conducted experiments to evaluate the order of all indicators, further validating the effectiveness of the proposed mutual information entropy-based measurement. Different from traditional boosting methods, which input all features simultaneously for prediction and primarily focus on optimizing performance by adjusting sample weights, emphasizing misclassified samples to improve overall accuracy, our model emphasizes the interrelationships between different medical test results for individual patients rather than assuming equal significance for all features. Furthermore, our approach focuses on dynamically selecting appropriate submodels for different data subsets, iteratively refining predictions by addressing the residuals of previous predictions. This tailored methodology allows for a more nuanced and adaptive prediction process, particularly suitable for complex medical datasets. It is important to note that our model was designed to predict the risk of developing hypoalbuminemia within 14 days of admission, rather than identifying the specific time point of its occurrence. As a result, the model was less sensitive to the time interval between the examination and the onset of hypoalbuminemia compared to models aimed at predicting specific time points. According to the results, the accuracy of the gradual fusion model achieved 95% in both cases and AUC were 0.96 and 0.9 respectively, which is higher than that of the single examine prediction and the early fusion prediction. Compared to the previous study, Our proposed model has a large improvement in the prediction effect. It indicated that the gradual fusion model proposed by us effectively utilizes the complementary information between different test data, at the same time, it also conforms to the practical diagnostic logic. Meanwhile we compared the predicted results with those without distinguishing pneumonia patients, the former results were much greater than the latter, which indicated the mechanism of hypoalbuminemia in patients with and without pneumonia was indeed different, separating them effectively reduces the impact of useless information when predicting hypoalbuminemia. Our proposed model can assist doctors to form an early prediction of geriatric patients who have just been admitted to hospital, give patients targeted diagnosis and treatment suggestions, slow down the deterioration of acute chronic diseases in geriatric patients, and thus reduce the risk of death.

Finally, we evaluated the importance of the four tests through sensitivity analysis of the gradual fusion model. The analysis results showed that the blood routine test results should be paid more attention to in patients with pneumonia complicated with hypoalbuminemia, while the urine routine test results should be paid more attention to in patients with non-pneumonia complicated with hypoalbuminemia. From a medical point of view, pneumonia is an infectious disease, when patients with hypoalbuminemia suffer from pneumonia, the cells in the blood will be greatly affected, such as the white blood cells in the blood will be greatly increased (47, 48). But patients with hypoalbuminemia without pneumonia are mainly affected by hypoalbuminemia, which will lead to reduced protein synthesis and increased excretion (42). This causes some indicators in the urine such as the amount of urine protein to cause large fluctuations.

**Figure 7:**
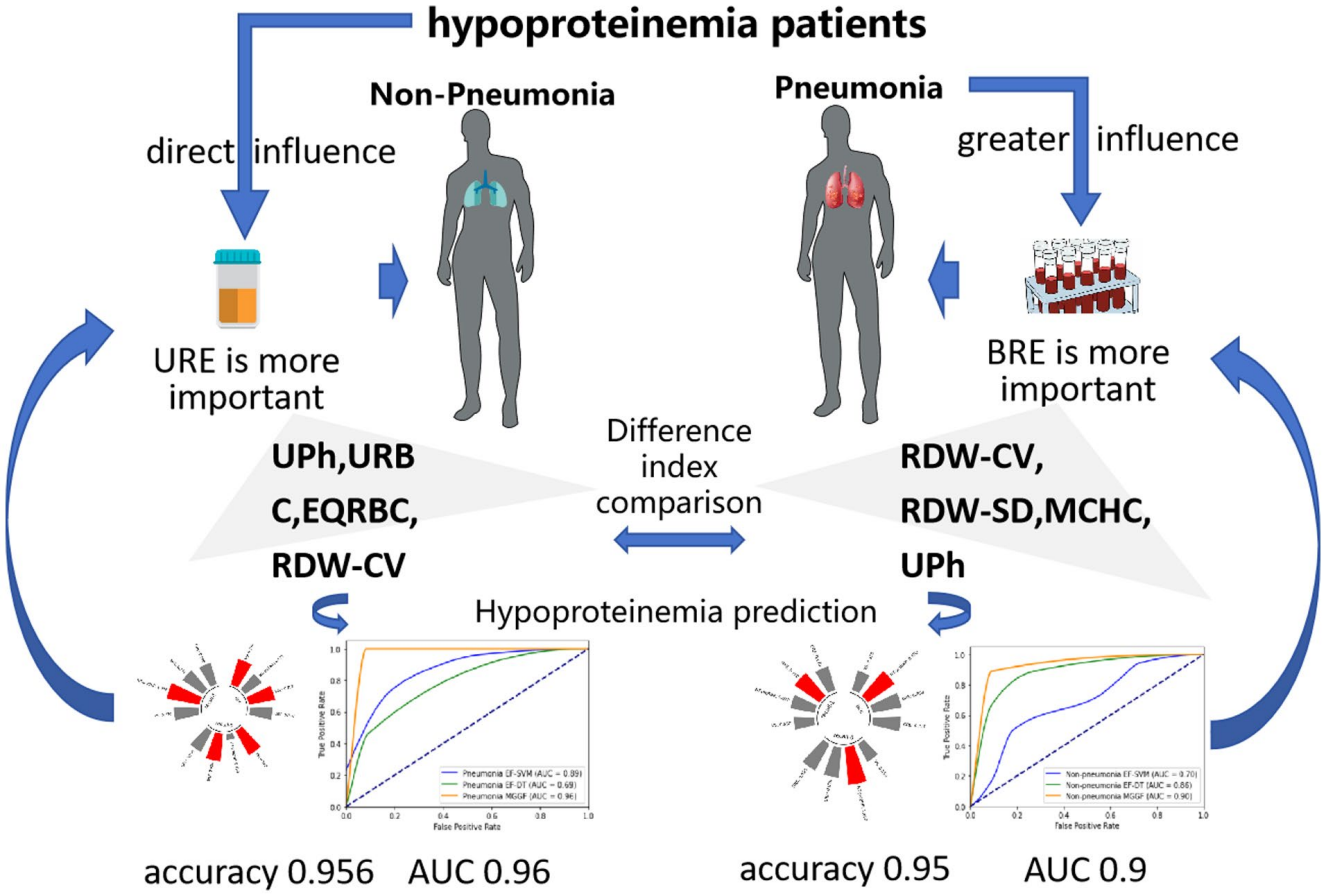
The main conclusions in our study. When patients with hypoalbuminemia suffer from pneumonia, the cells in the blood will be greatly affected, while hypoalbuminemia patients without pneumonia are mainly affected by hypoalbuminemia, which will lead to reduced protein synthesis and increased excretion. Our model sensitivity analysis also showed blood routine examine performs the best importance to predict hypoalbuminemia in pneumonia patients and urine routine examine performs the best importance to predict hypoalbuminemia in non-pneumonia patients, which were the same significance as clinical experience.

In future studies, we expect to obtain more cases and more derived test results to verify the validity of our model. In addition, we can also extend the model to the prediction of other diseases, hoping that the model can have a certain generalization and help more patients to carry out early prevention and treatment of diseases. The main conclusions can be seen in Figure 7.

## 5 Conclusion

We proposed a gradual fusion model to early predict hypoalbuminemia which improved the prediction accuracy by nearly 17.6% compared with the classical machine learning method. It can assist doctors to form an early prediction of geriatric patients who have just been admitted to hospital, for patients at high risk of developing hypoalbuminemia, frequent monitoring of serum protein levels is essential. Additionally, protein intake should be enhanced through oral nutritional supplements, intravenous nutrition, or parenteral nutrition. It is also important to avoid the overuse of diuretics or other medications that may contribute to protein loss. Clinical experience and model analysis jointly showed the changes in the blood of patients with hypoalbuminemia combined with pneumonia were more significant than that of patients with hypoalbuminemia alone, which was characterized by abnormal excretion due to low protein. We recommend that clinicians give particular attention to blood routine results when preventing hypoalbuminemia in patients with pneumonia, and controlling the underlying infection with appropriate antimicrobial therapy and managing fluid balance to avoid excessive fluid loss is crucial in mitigating the risk of hypoalbuminemia. For patients without pneumonia, more emphasis should be placed on urine routine examination results, measures such as optimizing renal function, addressing any underlying kidney diseases, and ensuring adequate hydration and nutrition are essential to prevent protein loss. By taking a comprehensive and proactive approach, clinicians can better manage and reduce the risk of hypoalbuminemia in both pneumonia and non-pneumonia patients.

## Data Availability

The raw data supporting the conclusions of this article will be made available by the authors, without undue reservation.
