# Supplementary material for: Construction of geriatric hypoalbuminemia predicting model for hypoalbuminemia patients with and without pneumonia and explainability analysis
Source: Front Med (Lausanne). 2024 Dec 31;11:1518222. doi: 10.3389/fmed.2024.1518222 (PMC11729439; doi:10.3389/fmed.2024.1518222)
Supplement: Supplementary file 1 [file Data_Sheet_1.docx]

Supplementary Material

# Mutual Information-Grey Relational Coefficient Gradual Fusion Model(MGGF) Construction Steps

1.Identify submodels for MGGF

Each step model of our proposed gradual fusion model is selected from a linear support vector machine, a support vector machine with radial basis function kernel, and a decision tree, which, like the model in the machine learning method, is also based on the Gini coefficient and a maximum depth of 4.

2.Determine the data entry sequence for MGGF

The average mutual information between the features and labels contained in each data is calculated, and the data is taken as the input of each step model according to the order of mutual information from small to large. The calculation method of mutual information is shown in formula (3).

$\boldsymbol{I(x,y)=}\sum_{\boldsymbol{x,y}} \boldsymbol{p(x,y)log}\frac{\boldsymbol{p(x,y)}}{\boldsymbol{p(x)p(y)}}$ (3)

Where $\mathbf{I(x,y)}$ denotes the mutual information of **x** and **y**, $\mathbf{p(x)}$ denotes the probability of $\mathbf{x=}\mathbf{x}_{\mathbf{i}}$ occurrence, $\mathbf{p(y}\mathbf{)}$ denotes the probability of $\mathbf{y=}\mathbf{y}_{\mathbf{i}}$ occurrence, and $\mathbf{p(x,y)}$ denotes the probability of $\mathbf{x=}\mathbf{x}_{\mathbf{i}}$ and $\mathbf{y=}\mathbf{y}_{\mathbf{i}}$ simultaneous occurrence.

3.Identify the submodels used for each step

The linear support vector machine is used as the first step, and the hypoproteinemia label is used as the label of the training model. Starting with the second step, the residual between the output of the previous step model and the label is calculated as the label of the currently trained model before determining the submodel currently in use. After determining the data to be used in the next step, the average grey association coefficient between the data and the residual label is calculated, using a linear support vector machine when the coefficient is between [0.8,1), using a support vector machine with radial basis function kernel when the coefficient is between [0.6,0.8), and using a decision tree when the coefficient is between [0,0.6).The core of the grey correlation coefficient is to determine the correlation degree between the feature sequence and the reference sequence by comparing their geometric similarity. The calculation method is shown in formula (4).

$\boldsymbol{c(x,y)=}\min_{\boldsymbol{i}} \frac{\boldsymbol{min|}\boldsymbol{x}_{\boldsymbol{i}}\boldsymbol{-}\boldsymbol{y}_{\boldsymbol{i}}\boldsymbol{|+\rho max|}\boldsymbol{x}_{\boldsymbol{i}}\boldsymbol{-}\boldsymbol{y}_{\boldsymbol{i}}\boldsymbol{|}}{\boldsymbol{max|}\boldsymbol{x}_{\boldsymbol{i}}\boldsymbol{-}\boldsymbol{y}_{\boldsymbol{i}}\boldsymbol{|+\rho max|}\boldsymbol{x}_{\boldsymbol{i}}\boldsymbol{-}\boldsymbol{y}_{\boldsymbol{i}}\boldsymbol{|}}$ (4)

4.Model stop condition

(1)The model stops growing when all data is used

(2)After the data and sub-models of each step are determined, the final prediction accuracy of the model is calculated according to formula (5).

$\left\{ \begin{aligned} \boldsymbol{\delta(}\bar{\boldsymbol{y}}\boldsymbol{,y) i=1} \\ \boldsymbol{\delta(}\boldsymbol{\gamma}_{\boldsymbol{1}}\boldsymbol{(}\bar{\boldsymbol{y}}\boldsymbol{,}\boldsymbol{\gamma}_{\boldsymbol{2}}\boldsymbol{(}\boldsymbol{\varepsilon}_{\boldsymbol{1}}\boldsymbol{,}\boldsymbol{\gamma}_{\boldsymbol{3}}\boldsymbol{(}\boldsymbol{\varepsilon}_{\boldsymbol{2}}\boldsymbol{,(...(}\boldsymbol{\gamma}_{\boldsymbol{i-1}}\boldsymbol{(}\boldsymbol{\varepsilon}_{\boldsymbol{i-2}}\boldsymbol{,}\boldsymbol{\varepsilon}_{\boldsymbol{i-1}}\boldsymbol{)))))),y) i>1} \end{aligned} \right.$ (5)

Where, $\boldsymbol{\delta(}\bar{\boldsymbol{y}}\boldsymbol{,y)}$ represents the proportion of the same elements of the output label and the real label calculated in the first step, that is, the accuracy rate. $\boldsymbol{\varepsilon}_{\boldsymbol{i-1}}$ indicates the residual label obtained in the i step, $\boldsymbol{\gamma}_{\boldsymbol{i-1}}\boldsymbol{(}\boldsymbol{\varepsilon}_{\boldsymbol{i-2}}\boldsymbol{,}\boldsymbol{\varepsilon}_{\boldsymbol{i-1}}\boldsymbol{)}$ indicating that the residual of the i step is used to correct the residual of the i-1 step until the output label of the first step is corrected. the article.

# Comparison of test differences between hypoproteinemia and non-hypoproteinemia patients

T test was used to test the difference in characteristics of HPs and NHPs, the results were shown in Supplementary table1. The table shows the significant difference level of each indicator. Three stars indicate P value ≤0.01, indicating a particularly significant difference between features; two stars indicate 0.01≤P value ≤0.05, indicating a significant difference between features; one star indicates 0.05≤P value ≤0.1, indicating a certain difference between features. It can be seen that the features with differences are concentrated in the blood routine and urine routine tests.

**Supplementary table1.** Feature difference between HPs and NHPs

| Feature | NHPs | HPs | p-value | Significance |
| --- | --- | --- | --- | --- |
| RDW-CV | 0.265±0.22 | 0.275±0.25 | 0.0001 | *** |
| RDW-SD | 0.277±0.19 | 0.222±0.22 | 0.0002 | *** |
| UPh | 0.272±0.27 | 0.458±0.32 | 0.0015 | *** |
| MCHC | 0.585±0.2 | 0.532±0.25 | 0.0148 | ** |
| HB | 0.534±0.24 | 0.489±0.25 | 0.0576 | * |
| RBC | 0.528±0.23 | 0.559±0.26 | 0.0830 | * |
| URBC | 0.039±0.15 | 0.035±0.17 | 0.0892 | * |
| EQRWC | 0.039±0.15 | 0.039±0.15 | 0.0892 | * |
| HCT | 0.45±0.24 | 0.526±0.26 | 0.1242 |  |
| MCV | 0.425±0.18 | 0.325±0.22 | 0.2082 |  |
| WBC | 0.161±0.14 | 0.168±0.17 | 0.2580 |  |
| NT-proBNP | 0.065±0.16 | 0.095±0.19 | 0.3593 |  |
| PLT | 0.345±0.16 | 0.481±0.25 | 0.6318 |  |
| RR | 0.38±0.15 | 0.542±0.21 | 0.6536 |  |
| UWBC | 0.041±0.15 | 0.09±0.22 | 0.6908 |  |
| EQWBC | 0.041±0.15 | 0.09±0.22 | 0.6909 |  |
| SBP | 0.522±0.21 | 0.504±0.2 | 0.7530 |  |
| USG | 0.317±0.22 | 0.294±0.23 | 0.8784 |  |
| MCH | 0.522±0.17 | 0.45±0.25 | 0.9361 |  |

Logistic regression was used to perform regression with each feature as independent variable and hypoproteinemia label (0,1) as dependent variable. The results were shown in Supplementary figure 2. In the figure, red shows the characteristics of VS, black shows the characteristics of NT-proBNP, green shows the BRE, and orange shows the characteristics of URE. As can be seen from the figure, the feature weights of blood routine and urine routine are significantly greater than those of the other two tests. Further through the weight coefficient of regression, we calculated that the average weight of the four tests for hypoproteinemia without distinguishing pneumonia was 0.1645, 0.042, 0.195, 0.51. The first two test data with the highest weight are urine routine and blood routine.


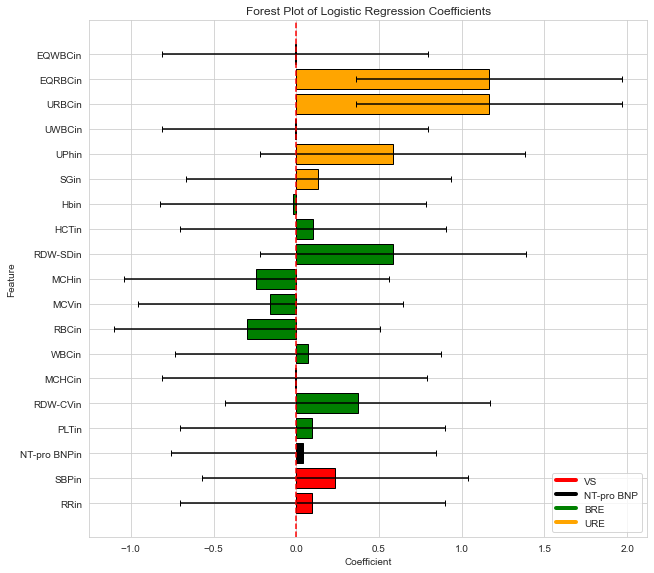


**Supplementary figure2**. logistic regression weight plot of HPs and NHPs

Pearson correlation coefficient and mantel test were used to analyze the correlation between features and the correlation between features and hypoproteinemia labels, and the results were shown in Supplementary figure 3. The size of the square in the figure indicates the correlation between the features, green indicates the positive correlation, and red indicates the negative correlation. The thickness of the line between the feature dot and the label dot in the upper right corner of the figure indicates the correlation between the feature and the label. Green indicates P value ≤0.01, and red indicates 0.01≤P value ≤0.05. As can be seen from the figure, the features significantly associated with hypoproteinemia were also distributed in blood routine and urine routine. The above analysis indicates that blood routine test and urine routine test are more important than the other two tests in evaluating hypoproteinemia.


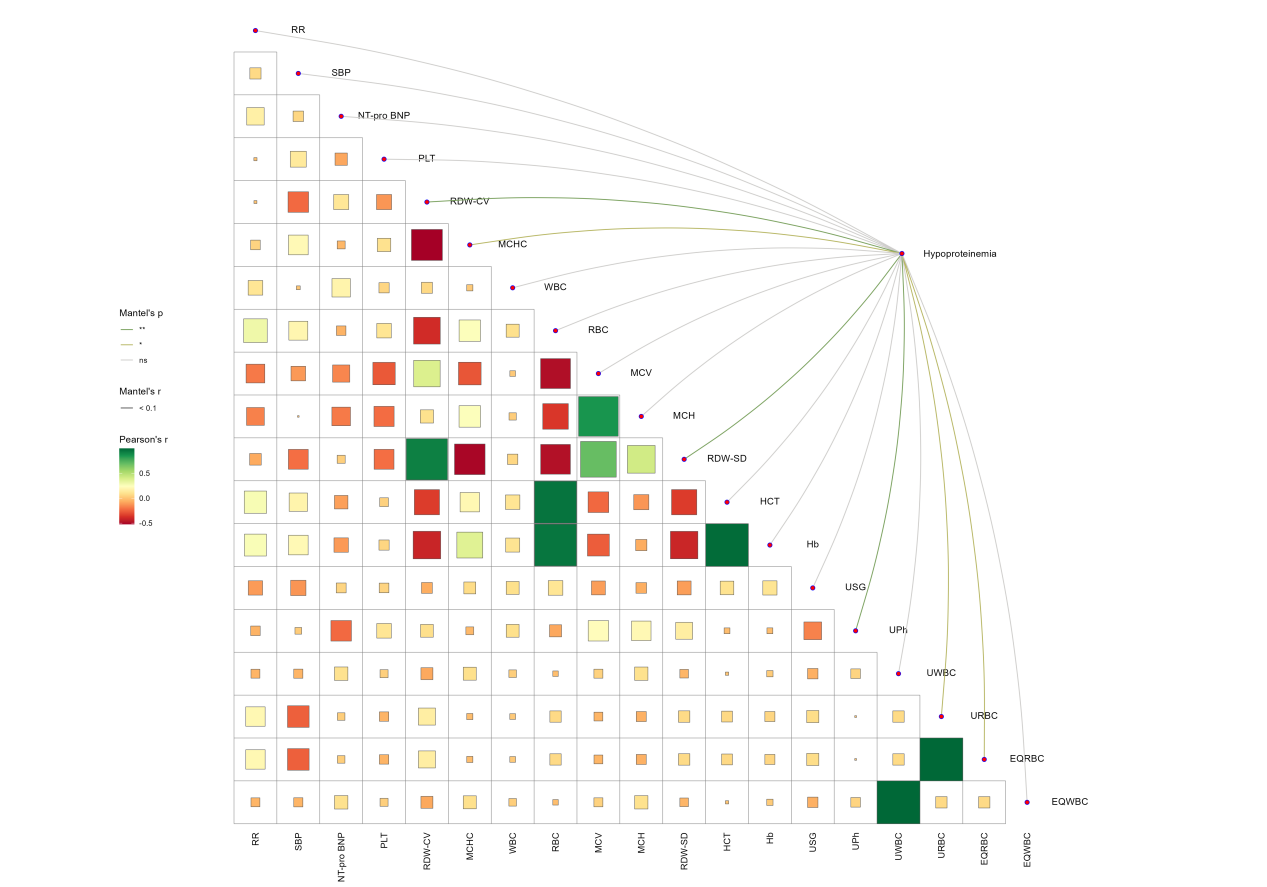


**Supplementary figure3.** Heat map of the correlation between features and labels of HPS and NHPs

## Mutual Information and Grey Correlation Coefficient When Constructing Gradual Fusion Model

**Supplementary table2.** Mutual information between the four tests and labels of all patients

| VS | NT-proBNP | BRE | URE |
| --- | --- | --- | --- |
| 0.07 | 0.036 | 0.065 | 0.018 |

**Supplementary table3.** Grey correlation coefficient and submodel from second step of MGGF for all patients

| Grey correlation coefficient | - | 0.674 | 0.583 | 0.585 |
| --- | --- | --- | --- | --- |
| submodel | Linear-SVM | RBF-SVM | DT | DT |

**Supplementary table4.** Mutual information between the four tests and labels of patients with and without pneumonia

|  | VS | NT-proBNP | BRE | URE |
| --- | --- | --- | --- | --- |
| patients with pneumonia | 0.114 | 0.023 | 0.099 | 0.012 |
| patients without pneumonia | 0.088 | 0.057 | 0.082 | 0.024 |

**Supplementary table5.** Grey correlation coefficient and submodel from second step of MGGF for patients with and without pneumonia

| patients with pneumonia | - | 0.657 | 0.572 | 0.539 |
| --- | --- | --- | --- | --- |
|  | Linear-SVM | RBF-SVM | DT | DT |
| patients without pneumonia | - | 0.6 | 0.588 | 0.548 |
|  | Linear-SVM | RBF-SVM | DT | DT |

1. **The Gradual Fusion Prediction Results of 24 Input Orders in All Patients**

we compared the gradual fusion prediction results of all 24 input orders of the four test data, as shown in Supplementary figure6. The red points represents the highest result in all results. It can be seen that the gradual fusion prediction based on mutual information-grey correlation coefficient has the best effect.


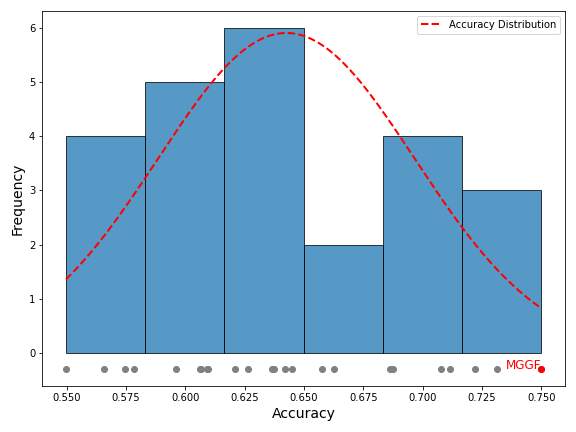


**Supplementary figure6.** The gradual fusion prediction accuracy based on mutual information-grey correlation coefficient and random input data
