# Supplementary material for: Construction of geriatric hypoalbuminemia predicting model for hypoalbuminemia patients with and without pneumonia and explainability analysis
Source: Front Med (Lausanne). 2024 Dec 31;11:1518222. doi: 10.3389/fmed.2024.1518222 (PMC11729439; doi:10.3389/fmed.2024.1518222)
Supplement: Supplementary file 2 [file Supplementary_file_1.docx]

# Appendix

TableA1 Indicators difference of PHPs and PNHPs

| Examine | Indicators | PNHPs | PHPs | p-value | significance^a^ |
| --- | --- | --- | --- | --- | --- |
| VS | SBP | 0.512 ± 0.26 | 0.482 ± 0.23 | 0.7691 |  |
|  | RR | 0.386 ± 0.23 | 0.595 ± 0.24 | 0.8852 |  |
| NT-proBNP | NT-proBNP | 0.233 ± 0.3 | 0.136 ± 0.24 | 0.7262 |  |
| BRE | RDW-CV | 0.297±0.27 | 0.332±0.27 | 0.0136 | ** |
|  | RDW-SD | 0.281±0.29 | 0.282±0.25 | 0.0297 | ** |
|  | MCHC | 0.532 ± 0.25 | 0.502 ± 0.28 | 0.0708 | * |
|  | MCV | 0.433 ± 0.25 | 0.376 ± 0.25 | 0.2514 |  |
|  | RBC | 0.524 ± 0.28 | 0.537 ± 0.3 | 0.2633 |  |
|  | Hb | 0.478 ± 0.3 | 0.471 ± 0.28 | 0.2763 |  |
|  | HCT | 0.486 ± 0.29 | 0.520 ± 0.30 | 0.4064 |  |
|  | WBC | 0.325 ± 0.24 | 0.193 ± 0.22 | 0.5867 |  |
|  | MCH | 0.496 ± 0.22 | 0.473 ± 0.26 | 0.6933 |  |
|  | PLT | 0.324 ± 0.21 | 0.478 ± 0.3 | 0.9927 |  |
| URE | UPh | 0.286±0.33 | 0.437±0.34 | 0.0414 | ** |
|  | USG | 0.311 ± 0.27 | 0.317 ± 0.27 | 0.5412 |  |
|  | UWBC | 0.071 ± 0.22 | 0.101 ± 0.22 | 0.2975 |  |
|  | EQWBC | 0.071 ± 0.22 | 0.101 ± 0.22 | 0.2976 |  |
|  | URBC | 0.106 ± 0.27 | 0.053 ± 0.22 | 0.3648 |  |
|  | EQRBC | 0.106 ± 0.27 | 0.053 ± 0.22 | 0.3648 |  |

a: ‘**’ represented significant and ‘*’ represented a little significant.

TableA2 Indicators difference of NPHPs and NPNHPs

| Examine | Indicators | NPNHPs | NPHPs | p-value | significance^a^ |
| --- | --- | --- | --- | --- | --- |
| VS | RR | 0.439 ± 0.15 | 0.578 ± 0.37 | 0.5582 |  |
|  | SBP | 0.456 ± 0.24 | 0.457 ± 0.27 | 0.9788 |  |
| NT-proBNP | NT-proBNP | 0.031 ± 0.13 | 0.183 ± 0.27 | 0.8258 |  |
| BRE | RDW-CV | 0.244 ± 0.21 | 0.262 ± 0.27 | 0.0536 | * |
|  | RDW-SD | 0.293 ± 0.17 | 0.339 ± 0.34 | 0.1271 |  |
|  | PLT | 0.36 ± 0.21 | 0.405 ± 0.29 | 0.1533 |  |
|  | Hb | 0.540 ± 0.23 | 0.521 ± 0.3 | 0.2460 |  |
|  | HCT | 0.539 ± 0.25 | 0.554 ± 0.31 | 0.2830 |  |
|  | RBC | 0.554 ± 0.24 | 0.532 ± 0.35 | 0.3523 |  |
|  | MCHC | 0.595 ± 0.2 | 0.47 ± 0.32 | 0.4578 |  |
|  | MCH | 0.571 ± 0.17 | 0.528 ± 0.3 | 0.4679 |  |
|  | MCV | 0.466 ± 0.17 | 0.405 ± 0.27 | 0.7275 |  |
|  | WBC | 0.151 ± 0.14 | 0.474 ± 0.3 | 0.8628 |  |
| URE | UPh | 0.284 ± 0.27 | 0.587 ± 0.38 | 0.0126 | ** |
|  | URBC | 0.033 ± 0.13 | 0.123 ± 0.29 | 0.0133 | ** |
|  | EQRBC | 0.033 ± 0.13 | 0.123 ± 0.29 | 0.0133 | ** |
|  | USG | 0.324 ± 0.21 | 0.41 ± 0.25 | 0.5548 |  |
|  | UWBC | 0.037 ± 0.14 | 0.155 ± 0.31 | 0.6674 |  |
|  | EQWBC | 0.037 ± 0.14 | 0.155 ± 0.31 | 0.6675 |  |

a: ‘**’ represented significant and ‘*’ represented a little significant.
